# Heterozygous mutations in *valosin*-*containing protein* (*VCP*) and resistance to VCP inhibitors

**DOI:** 10.1038/s41598-019-47085-9

**Published:** 2019-07-29

**Authors:** Prabhakar Bastola, Rabeya Bilkis, Cristabelle De Souza, Kay Minn, Jeremy Chien

**Affiliations:** 1 Department of Laboratory Medicine, University of California San Francisco, 185 Berry Street, San Francisco, 94143 California USA; 2 Division of Molecular Medicine, University of New Mexico Health Sciences Center, 915 Camino de Salud NE, Albuquerque, 87131 New Mexico USA; 3 Department of Biochemistry and Molecular Medicine, University of California, Davis. 2700 Stockton Blvd. Sacramento, 95817 California, USA; 4 Department of Laboratory Medicine and Pathology, Mayo Clinic, 200 First Street SW, Rochester, 55905 Minnesota USA; 5 Department of Obstetrics and Gynecology, University of California, Davis, 4860 Y Street, Suite 2500 Sacramento, CA USA

**Keywords:** Ovarian cancer, Cancer therapeutic resistance

## Abstract

In recent years, multiple studies including ours have reported on the mechanism of resistance towards valosin-containing protein (VCP) inhibitors. While all these studies reported target alterations via mutations in *VCP* as the primary mechanism of resistance, discrepancies persist to date regarding the zygosity of these mutations responsible for the resistance. In addition, the extent to which resistant cells harbor additional mutations in other genes is not well described. In this study, we performed global transcript analysis of the parental and previously reported VCP inhibitor (CB-5083) resistant cells and found additional mutations in the resistant cells. However, our CRISPR-Cas9 gene editing studies indicate that specific mutations in *VCP* are sufficient to produce resistance to CB-5083 suggesting the importance of on-target mutations in VCP for resistance. Strikingly, our analysis indicates a preexisting heterozygous frameshift mutation at codon 616 (N616fs*) in one of the *VCP* alleles in HCT116 cells, and we showed that this mutant allele is subjected to the nonsense-mediated decay (NMD). Accordingly, we identified a heterozygous mutation at codon 526 (L526S) in genomic DNA sequencing but a homozygous L526S mutation in complementary DNA sequencing in our independently generated CB-5083 resistant HCT116 cells, implying that the L526S mutation occurs in the allele that does not harbor the frameshift N616fs* mutation. Our results suggest the NMD as a possible mechanism for achieving the homozygosity of VCP mutant responsible for the resistance to VCP inhibitors while resolving the discrepancies among previous studies. Our results also underscore the importance of performing simultaneous genomic and complementary DNA sequencing when attributing mutational effects on the functionality particularly for an oligomer protein like VCP.

## Introduction

Valosin-containing protein (VCP), also known as p97, has been shown to be involved in a myriad of cellular functions associated with the protein quality control mechanisms including the endoplasmic reticulum-associated degradation (ERAD)^1^, mitochondria-associated degradation^2^, the ubiquitin proteasome system (UPS)^3^, chromatin-associated degradation^4^ and autophagy^5–7^. VCP forms a homo-hexameric structure mediating such diverse functions through its association with a network of cofactors proteins (p47, Ufd1-Npl4, p37, etc.)^8^. The barrel shaped VCP extracts ubiquitylated substrates facilitating downstream degradation primarily through the UPS; however, several studies have now unearthed the role of VCP in the clearance of damaged mitochondria^9^ and damaged lysosome^10^ through lysosomal mediated degradation.

Non-oncogenes, including components of the protein quality control pathway, have been proposed as therapeutic targets for cancer therapy^11,12^. This led to an interest in the inhibition of VCP, a common essential protein in cancer, since such inhibition would result in increased proteotoxic stress and subsequent cell death in cancer^13^. Several efforts towards the development of VCP inhibitors^14,15^ led to the first-in-class oral VCP inhibitor, CB-5083^16,17^. Multiple studies have now shown pre-clinical efficacy of CB-5083 in several hematological malignancies^16,18^ and solid tumor models^16,19^.

With significant clinical interest in the development of VCP inhibitors^16–18^, multiple studies including ours, have evaluated the resistance mechanisms towards different classes of VCP inhibitors^16,20^. Current literature regarding resistance to VCP inhibitors shows that missense mutations in the coding region of *VCP* are the primary mechanism of resistance; however, to the best of our knowledge none of these studies have focused on the transcriptomic effects of such mutations. In this study, we conducted a comprehensive RNA sequencing analysis as opposed to targeted sequencing of *VCP* as reported in previous studies^16,20,21^. Here, we report the presence of additional mutations in resistant cells. Next, using homology-directed repair (HDR) mediated CRISPR-Cas9 based gene editing, we show conclusively that specific mutations in *VCP* are sufficient to produce resistance to CB-5083.

Discrepancies persist in current literature regarding the zygosity of *VCP* mutations upon treatment with VCP inhibitors. Initial studies by Anderson *et al*. reported that homozygous missense mutations in *VCP* are sufficient to produce resistance to the VCP inhibitor CB-5083 in HCT116 cells^16^. Subsequently, Her *et al*. reported that a single heterozygous missense mutation in *VCP* (A530T) is sufficient to produce resistance to the VCP inhibitor NMS-873 in HCT116 cells^20^. Her *et al*. speculated that such a difference in zygosity may reflect the potential differences in the mechanisms of action between CB-5083 and NMS-873. However, in our previous study, we successfully generated OVSAHO cells with resistance to both CB-5083 and NMS-873 inhibitors that possess unique patterns of co-selected mutations with heterozygous missense mutations in one *VCP* allele and heterozygous nonsense mutations in the other *VCP* allele^21,22^.

The concept of mutated VCP being dominant over wild-type VCP is supported by clinical evidence where heterozygous mutations in *VCP* are found in patients with multisystem proteinopathy^8^. Given that VCP forms a functional hexamer for various protein quality control and vesicular-damage response function, it is not surprising that a single mutant-VCP monomer may compromise the function of the hexamer leading to progressive proteinopathy and associated diseases. However, such a mutant-dominant concept may not be applicable in resistance to VCP inhibitors. Given that VCP forms a functional hexamer, it is conceivable that in a hexamer consisting of both wild-type and mutated VCP subunits, although mutant subunits may be resistant to VCP inhibitors, wild-type subunits will be sensitive and therefore could be inhibited. In this scenario, VCP inhibitors may compromise the function of the hexamer by selectively inhibiting the wild-type subunits. Based on this concept, we posit that cells resistant to VCP inhibitors have homozygous mutations. Given prior contradictory reports regarding the role of *VCP* zygosity in the resistance, we made an elaborate attempt to reach a consensus. In this study, we report a preexisting heterozygous frameshift mutation (N616fs*) in HCT116 cells, the cell line used in prior studies. We show this frameshift mutation in HCT116 cells is subject to nonsense-mediated decay (NMD). Additionally, we generated independent HCT116 clones that are resistant to CB-5083 and show that these resistant cells harbor mutations in *VCP* in the allele that does not have the preexisting frameshift mutation. Our results help to explain the discrepancies between previous studies as well as highlight the importance of sequencing both the genomic DNA (gDNA) and the complementary DNA (cDNA) when evaluating mutations in *VCP* associated with resistance to VCP inhibitors.

## Materials and Methods

### Cell lines and cell culture

Establishment of OVSAHO resistance cells (OVSAHO-R1) is described in our previous publication^21^. OVSAHO parental, OVSAHO-R1, HEK293T parental and HEK293T-HDR edited cells were cultured in RPMI (VWR, L0105-0500) with 10% fetal bovine serum (Sigma-Aldrich, F8067), and 1% penicillin-streptomycin (VWR, 97063-708). HCT116 cells (HCT116-par) were purchased from the American Type Culture Condition (ATCC, CCL-247). HCT116-resistant cells (HCT116-res) were generated through ten rounds of intermittent dosing with CB-5083 (Selleckchem, S8101) for 24 hours followed by a recovery phase in the drug-free medium for 5–10 days. CB-5083 treatment started at 2.5 *µ*M and with every round, the concentration of CB-5083 was increased by 0.5 *µ*M. Both HCT116-par and HCT116-res were cultured in McCoy’s 5a Medium (ATCC, 30–2007) with 10% fetal bovine serum (FBS). All cells were incubated in a humidified incubator with 5% CO_2_ at 37 °C and were periodically checked for mycoplasma contamination.

### CRISPR-Cas9 mediated homology-directed repair

To perform CRISPR-Cas9 mediated homology-directed repair (HDR), E470K and E470D targeting guide RNA, HDR templates and Cas9 expression plasmid were provided by Genome Engineering and iPSC Center (GEiC), Saint Louis. To create specific CRISPR-knock ins, Cas9 was transiently expressed through a Cas9 expression plasmid, while targeting guide RNA (ACTGCGGGAAACCGTGGTAG) and HDR repair templates were provided as RNA and single strand DNA oligos, respective. HDR templates were designed to harbor a specific point mutation with an additional silent mutation. For transfection, 0.5 × 10^6^ HEK-293T cells were plated in RPMI +10% FBS and were allowed to incubate overnight in a humidified incubator with 5% CO_2_ at 37 °C. Next day, the Cas9 expression plasmid, guide RNA and the respective HDR template were transfected using EndoFectin Max (GeneCopoeia, EF014) according to the manufacturer’s protocol. Forty-eight hours later, media was aspirated, and cells were treated with 2.5 *µ*M CB-5083 for 72 hours. Cells were then allowed to recover. Single cell colonies were picked and expanded. Sanger sequencing using our previously published primers provided confirmation of the CRISPR-Cas9 mediated HDR repairs^21^.

### Chemicals, SRB assay and colony formation assay

CB-5083 (Selleckchem, S8101) and NMS-873 (Selleckchem, S7285) were dissolved in DMSO to make a 50 mM stock solution. Sulforhodamine B (SRB) assay was used to determine the cell viability following the treatment with CB-5083 and NMS-873. Similarly, colony formation assay was used to determine the long-term survival following the treatment with CB-5083. Both SRB and colony formation assay were performed according to our previously established protocols^19,21^.

### Genomic and RNA extraction, PCR amplification, and sanger sequencing

Genomic and RNA extraction, PCR amplification, and sequencing were performed according to our previously established protocols^21^. We used the forward primer TAATGGAGGGGATGCTTCTG and the reverse primer GCCCTCAGGCAAATCAATAC to amplify the *VCP* exon 13, the forward primer CATGCTGGTTTCGGATTTCT and the reverse primer GCCTGAGGACTCATGCAAGT to amplify the *VCP* exon 14, and the forward primer ATGAGCTAGATGCCATCGCT and the reverse primer AAGTCCACATCCTTGGCAAC for the complementary DNA sequencing. For exogenous VCP quantification, we used the forward primer AGCAAGGGCGAGGAGCTGTTC and the reverse primer TAGCGGCTGAAGCACTGCACG from eGFP sequence. As a loading control, we used the GAPDH forward primer GAAACTGTGGCGTGATGGC and the reverse primer CACCACTGACACGTTGGCAG. For VCP cDNA sequencing in NMD studies, we used the forward primer GTGGTAGAGGTGCCACAGGT and the reverse primer CTGAGGATGGCAGGATCAAT. All primers are listed in 5′-3′ direction. Sequencing results can be found in https://osf.io/uzdbw/.

### RNA-sequencing analysis

OVSAHO parental and resistant cells were seeded in 6-well plates (0.5 × 10^6^ cells/well). The next day, cells were either treated with vehicle (DMSO) or 5 *µ*M CB-5083 for 6 hours. Each treatment condition was performed in three biological replicates. Cells were scraped and collected by centrifugation. Cells were then washed twice with phosphate-buffered saline (pH 7.0), and 1 ml Trizol reagent (Invitrogen, 15596–028) was added to each sample. Total RNA was extracted using the Trizol reagent according to the manufacturer’s protocol. One microgram of total RNA was used to prepare the sequencing libraries using Illumina TruSeq Stranded Total RNA Sample Preparation Kit based on the manufacturer’s protocol. RNA sequencing was performed using Illumina HiSeq. 4000 by the Genome Sequencing Core at Mayo Clinic, Rochester, Minnesota.

Sequences produced by Illumina sequencing in the FASTQ format were imported in CLCBio Genomics WorkBench (ver. 9) and mapped to the human reference genome (hg19). Reads mapping to annotated genes were then counted and differentially expressed genes between two groups were determined by DESeq. 2. False discovery rate (FDR) cutoff value of 0.001 and an absolute fold change of ≥1.5 were used to identify differentially expressed genes with high levels of statistical significance between two groups. To identify genes induced or suppressed by CB-5083, a separate analysis was performed in parental cells and CB-5083 resistant cells. For pathway analysis, differentially upregulated or downregulated genes were analyzed using the Metascape web tool^23^.

### Quantitative RT-PCR

Total RNA was extracted with Trizol reagent (Invitrogen, 15596–028) according to the manufacturer’s manual. The complementary DNA (cDNA) was synthesized using SuperScript II reverse transcriptase (Invitrogen, 180604014) with 1 μg of total RNA in a 20 μL reaction. The resulting cDNA was diluted 1:20 in nuclease-free water and 1 μL was used per qPCR reaction with three technical replicates per sample. qPCR was carried out using Power SYBR Green PCR Master Mix (Thermo Fisher Scientific, 4367659) on a CFX96 Real-Time PCR Detection System (Bio-Rad) including a non-template negative control. Amplification of *GAPDH* was used to normalize the level of mRNA expression. Primers used in the assay are shown in the Supplemental Table S3.

### Site-directed mutagenesis, plasmid preparation, and transient plasmid transfection

VCP (wt)-EGFP was a gift from Nico Dantuma (Addgene, 23971)^24^. 616 fs* mutation was generated by performing site-directed mutagenesis according to the QuickChange XL site-directed mutagenesis protocol (Agilent, Cat#200516). Primers sequences are forward: GCATGTCCACAAAAAAAATGTGTTCATCATTGGCGC and reverse: GCGCCAATGATGAACACATTTTTTTTGTGGACATGC. Primers are listed in 5′-3′ direction. Plasmids were prepared using Qiagen plasmid mini kit (Qiagen, 12123) based on the manufacturer’s protocol.

For transient plasmid transfection, 0.5 × 10^6^ HEK293T cells/well were seeded in 6-well plates in RPMI media with 10% fetal bovine serum without penicillin & streptomycin. Cells were then incubated overnight in a 37 °C humidified incubator containing 5% CO_2_. The following day, plasmids were transfected using EndoFectin Max (GeneCopoeia, EF014) based on the provided manufacturer’s protocol. Forty-eight hours later, media was aspirated and cells were collected for genomic DNA, RNA and protein extraction.

### Cell proliferation assay

For the proliferation assay, 0.5 × 10^5^ cells per well of HCT116 parental and HCT116 resistant cells were plated in 6-well plates. At Day 1, 2, 3 and 4, cells were trypsinized, harvested and total number of live cells were counted using the Countess^TM^ II Automated Cell Counter (Invitrogen). Each time point represents cells plated in three independent replicates.

### Western blot

0.5 × 10^6^ cells were plated in 6 well plates. Next day, cells were treated with the appropriate compound or vehicle (DMSO). Cells were harvested at different time points or treated at different concentrations according to the experimental design. To harvest, cells were scrapped, centrifuged and washed with 1X phosphate buffered saline (PBS) pH 7.0. Cells were then lysed with an equal volume of lysis buffer: 2X Laemmli Buffer (BioRad, 1610737) + 5% 2-Mercaptoethanol (Sigma-Aldrich, M3148) by heating at 95 °C for 10 minutes. Equal volumes of samples were loaded in SDS-PAGE and electroblotted onto polyvinylidene difluoride (PVDF) membranes. Blotted membranes were incubated in 3% fetal bovine serum albumin (BSA) prepared in Tris-buffered saline with 0.5% Tween-20 (TBS-T) solution for 1 hour at room temperature and were incubated with appropriate primary antibodies prepared in 3% BSA in TBS-T solution overnight at 4 °C. β-actin antibody was incubated for 1 hour at room temperature. Membranes were then washed with TBS-T solution and incubated with appropriate horseradish peroxidase (HRP)-conjugated secondary antibodies prepared in 3% BSA in TBS-T solution for 1 hour at room temperature. Immunocomplexes on the blots were visualized by ThermoScientific Dura chemiluminescent substrate (Fisher, PI34076) or Femto chemiluminescent substrate (Fisher, PI34096) and recorded by Bio-Rad imager (ChemiDoc^TM^ MP Imaging System).

Primary antibodies used for Western blotting included VCP (Santa Cruz Biotechnology, 20799), PERK (Cell Signaling, 3192 S), phospho-eIF2α (Abcam, ab32157), CHOP (Cell Signaling, 5554 S), s-XBP1 (Cell Signaling, 12782), Grp78 (Cell Signaling, 3177 S), Ub-48 (Sigma-Aldrich, 05–1307), GFP (Santa Cruz Biotechnology, 9996), and β-actin (Sigma-Aldrich, A1978). Secondary antibodies included HRP-linked anti-rabbit IgG (Cell Signaling, 2368), and HRP-linked anti-mouse IgG (Cell Signaling, 7076).

## Results

### Transcriptomic analysis indicates the endoplasmic reticulum (ER) stress pathway is activated in both resistant and parental cells

Previous studies have shown that the mechanisms of action pertaining to drugs can be inferred from the perturbed transcriptomes induced by the drugs^25^. To investigate potential molecular mechanisms contributing to CB-5083 resistance, we characterized the transcriptomes perturbed by CB-5083 in previously reported OVSAHO parental and OVSAHO resistant cells (OVSAHO-R1)^21^. We performed RNA sequencing of parental OVSAHO and CB-5803-resistant OVSAHO-R1 after treatment with vehicle (DMSO) or CB-5083 (5 *µ*M) for 6 hours. We selected the 6-hour treatment duration to limit the perturbation of gene expression to the primary response induced by CB-5083, as described in previous studies^25^.

**Figure 1:**
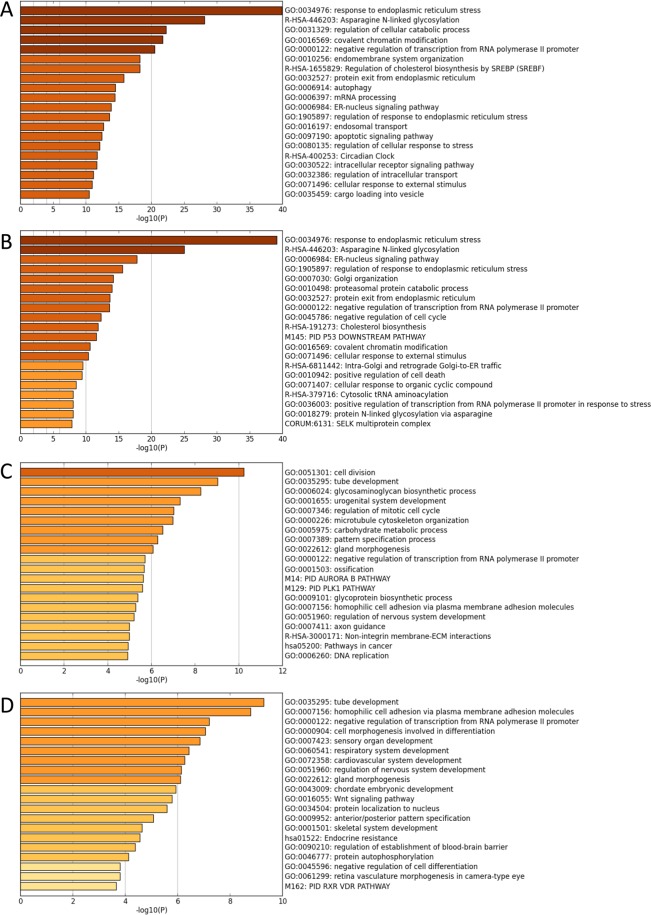
Differential regulation of pathways with CB-5083 treatment. (**A**) Inferred pathways upregulated by 5 *µ*M of CB-5083 treatment in the OVSAHO parental cells. (**B**) Inferred pathways upregulated by 5 *µ*M of CB-5083 treatment the resistant (OVSAHO-R1) cells. (**C**) Inferred pathways associated with genes downregulated by 5 *µ*M of CB-5083 treatment in OVSAHO parental cells. (**D**) Inferred pathways associated with genes downregulated by 5 *µ*M of CB-5083 treatment in the resistant (OVSAHO-R1) cells. Terms with the prefix “GO” are from the Gene Ontology Consortium, the prefix “R-HSA” from the Reactome, the prefix “M” from the Gene Set Enrichment database, and the prefix “hsa” is from the KEGG database.

The Metascape analysis of differentially expressed transcripts between the vehicle and CB-5083-treated samples within each cell line indicated the endoplasmic reticulum stress (ER stress) response pathway to be the most activated pathway in both cell lines (Fig. 1A,B). Similarly, pathways pertaining to the endoplasmic reticulum and protein homeostasis were activated in both cell lines. In parental cells, the Metascape analysis identified autophagy and apoptosis signaling pathways to be upregulated (Fig. 1A). Such pathway activation can be attributed to the induction of the unfolded protein response (UPR) mediated apoptosis^19^ and autophagy^16^. Additionally, in parental cells, genes associated with cell division, Aurora B pathway, PLK1 pathway, DNA replication, and pathways in cancer are downregulated by CB-5083 (Fig. 1C). In resistant cells, genes associated with the Wnt signaling pathway are downregulated by CB-5083 (Fig. 1D). The complete results of the Metascape analysis are available at the following link: https://osf.io/uzdbw/.

**Figure 2:**
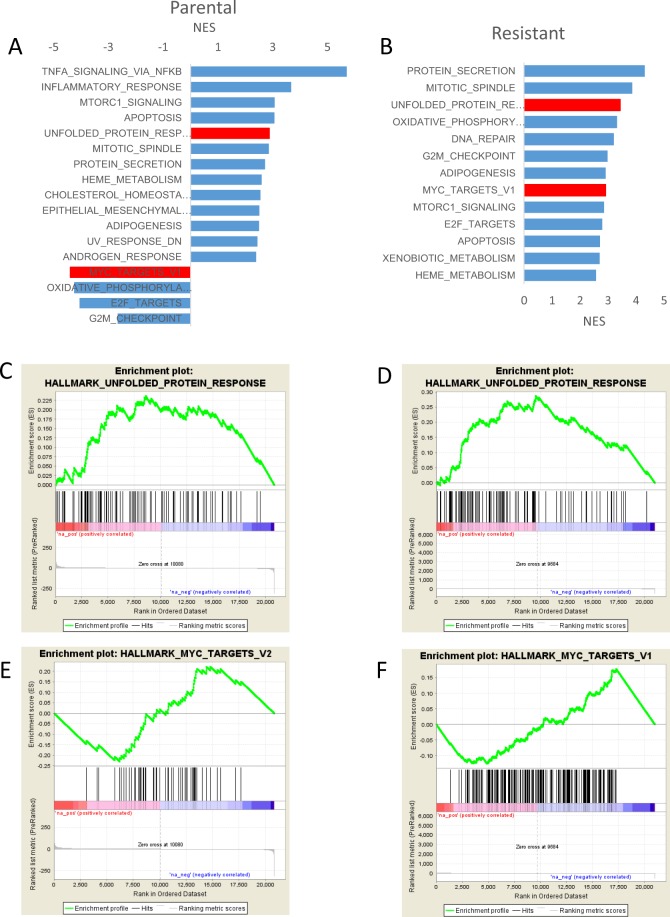
The Gene Set Enrichment Analysis indicates unfolded protein response gene set is positively enriched in CB-5083-treated cells. The complete results of GSEA analysis are available at the following link: https://osf.io/uzdbw/. GSEA analysis showing the gene set enrichment analyses in parental (**A**) and resistant (**B**) cell line with CB-5083 treatment. Enrichment plots depicting the enrichment pattern in the unfolded protein response (UPR) pathway in parental (**C**) and resistant (**D**) cells with CB-5083 treatment. Enrichment plots depicting the enrichment pattern in the myc targets in parental (**E**) and resistant (**F**) cells with CB-5083 treatment.

Next, we performed the Gene Set Enrichment Analysis (GSEA) in both our cell lines. GSEA identifies hallmark gene sets for TNF*α* signaling, inflammatory response, mTORC1 signaling, apoptosis, the UPR, and genes downregulated in response to UV radiation were positively enriched in CB-5083-treated parental cells compared to DMSO-treated cells at FDR q-value < 0.001 (Fig. 2A). Additionally, the hallmark gene sets for MYC targets, E2F targets, and DNA repair were negatively enriched in CB-5083 treated parental cells (Fig. 2A). These results suggest that MYC, E2F, and DNA repair pathways are inhibited by CB-5083. In the resistant cells, the GSEA identifies hallmark gene sets for the UPR, DNA repair, MYC targets, E2F targets, mTORC1 signaling, apoptosis, and genes downregulated in response to UV radiation were positively enriched in CB-5083-treated cells compared to DMSO treated cells at the FDR q-value < 0.001 (Fig. 2B). No gene sets were negatively enriched in CB5083-treated resistant cells (Fig. 2B).

Our results indicate that, although the UPR gene sets are positively enriched in both groups (Fig. 2A–D), the gene set comprising of MYC targets is negatively enriched in parental cells treated with CB-5083, whereas it is positively enriched in resistant cells treated with CB-5083 (Fig. 2E,F). Similarly, oxidative phosphorylation, E2F targets and G2M checkpoint gene sets were negatively enriched in parental cells treated with CB-5083, whereas they are positively enriched in resistant cells treated with CB-5083 (Fig. S1).

### The transcriptomic response is more pronounced in parental cells than in resistant cells

**Figure 3:**
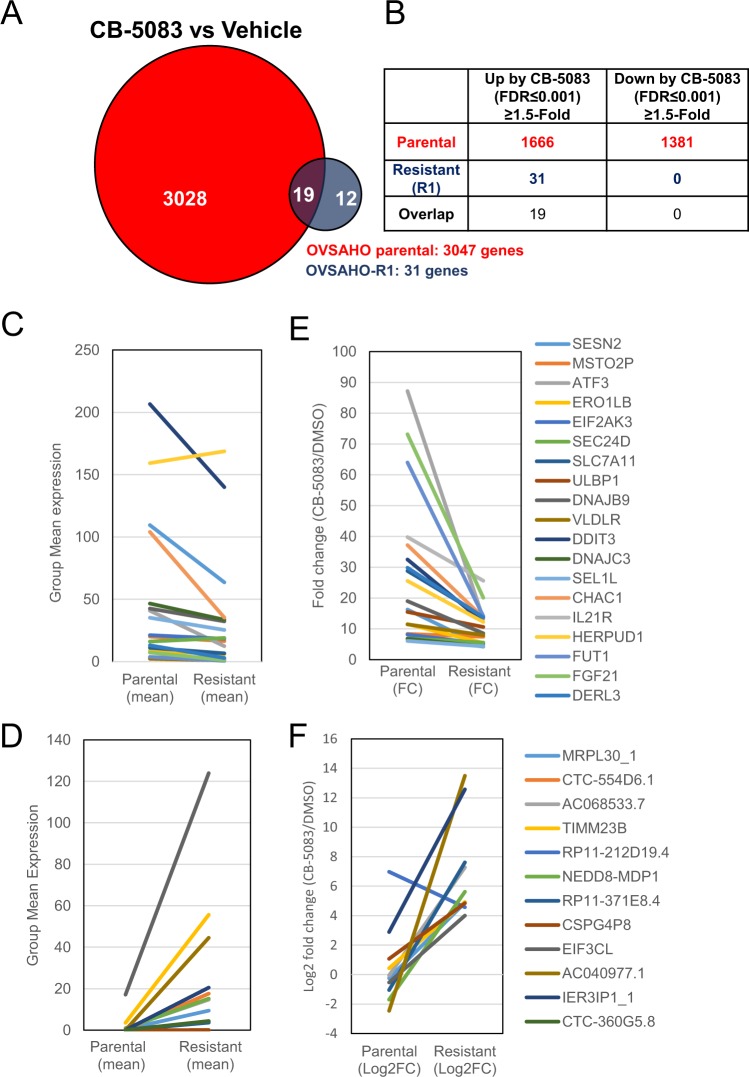
The number of differentially expressed transcripts with CB-5083 treatment. (**A**) The Venn diagram represents the number of differentially regulated transcripts in both parental and resistant cell lines upon 6 hours of treatment with CB-5083 (5 *µ*M). Differential expression was calculated based on the false discovery rate ≤0.001 and fold change ≤1.5. (**B**) The table indicates the number of transcripts that are differentially regulated (upregulated and downregulated) in OVSAHO-parental and OVSAHO-R1 cells by CB-5083 treatment. (**C**,**D**) Plots represent maximum mean expression values in 19 shared transcripts that were upregulated with CB-5083 treatment in OVSAHO parental and resistant cells (**C**) and 11 transcripts with higher expression in resistant cells treated with CB-5083 (**D**). (**E**,**F**) Relative log2 fold change values of the same transcripts as shown in C and D. Each line represents a transcript.

The analysis of the transcriptome perturbed by CB-5083 in parental and resistant cells shows a profound change in parental cells compared to resistant cells. A total of 3047 transcripts were differentially expressed in the parental cells after CB-5083 treatment, while only 31 transcripts were differentially expressed in OVSAHO-R1 after CB-5083 treatment, with an overlap in 19 transcripts between the two cell lines (Fig. 3A). In parental cells, 1666 genes were upregulated, and 1381 genes were downregulated at 1.5-fold or more by CB-5083 at the false discovery rate (FDR) ≤ 0.001 (Fig. 3A,B). In contrast, only 31 genes were upregulated by at least 1.5-fold at the FDR ≤ 0.001 in resistant cells by CB-5083. No genes were significantly downregulated by CB-5083 in resistant cells (Fig. 3B). Nineteen genes were commonly upregulated by CB-5083 in both cell lines (Fig. 3B).

Next, we focused on the 19 shared transcripts that were upregulated with CB-5083 in both cell lines. We plotted the upregulated transcripts based on their maximum mean expression values (Fig. 3C, left panel) and relative fold change values (Fig. 3E, right panel). Based on the maximum mean expression values, DDIT3 (CHOP) was the most highly upregulated transcript in the parental group (Fig. 3C). Likewise, based on the fold change values, ATF3 was the most highly upregulated transcript among the parental group (Fig. 3E). Considering that *CHOP* and *ATF3* are upregulated upon the induction of the UPR, our results further confirm that CB-5083 induces a strong UPR signature. Although resistant cells show induction of these transcripts, the maximum mean expression values for DDIT3 (CHOP) as well as fold change for ATF3 transcript are lower in the resistant cell line (OVSAHO-R1) than parental cells, suggesting that the effect of CB-5083 is consistently attenuated in the resistant cells. We validated the difference in CHOP upregulation between parental and resistant cells upon CB-5083 treatment by quantitative reverse transcription-PCR (qRT-PCR) (Fig. S3). Similarly, we analyzed the 12 transcripts that were upregulated only in OVSAHO-R1. These included transcripts such as IER3IP1_1, EIF3CL, and TIMM23B (Fig. 3D,F). IER3IP1 upregulation in resistant cells upon CB-5083 treatment was further confirmed by qRT-PCR (Fig. S3). Upregulation of these transcripts may represent an adaptive response that allows OVSAHO-R1 to escape CB-5083-mediated cytotoxic effect.

### The Connectivity Map analysis demonstrates similarities between the CB-5083 mechanism and the mechanisms of protein synthesis inhibitors and proteasome inhibitors

**Figure 4:**
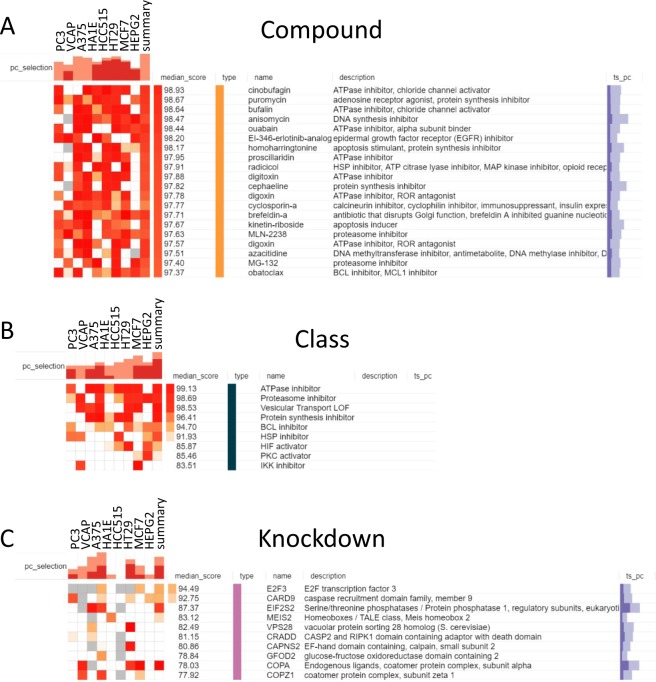
Connectivity Map analysis indicates CB-5083 treatment produced perturbed transcriptomic profile similar to ATPase inhibitors and protein synthesis inhibitors. (**A**) Similarities in perturbed transcriptomic profiles produced by CB-5083 and other compounds. (**B**) Similarities in perturbed transcriptomic profiles produced by CB-5083 and classes of compounds. (**C**) The similarity in perturbed transcriptomic profiles produced by CB-5083 and specific gene knockdown. The complete results of CMAP analysis are available at the following link: https://osf.io/uzdbw/.

The analysis of the transcriptome perturbed by CB-5083 in parental cells through the Connectivity Map web resources indicates that the perturbed transcriptome produced by CB-5083 is similar to the ATPase inhibitor cinobufagin as well as the protein synthesis inhibitor puromycin (Fig. 4A). Additional similarities are seen with proteasome and heat shock protein inhibitors, such as radicicol, MLN-2238, and MG-132. Since CB-5083 is a competitive ATPase inhibitor targeting the protein quality control mechanisms, these results further confirm that CB-5083 display the expected on-target effect considering that it induces the perturbed transcriptome similar to those induced by a class of drugs belonging to ATPase inhibitors, proteasome inhibitors, and protein synthesis inhibitors (Fig. 4B). CB-5083 treatment also produces a perturbed transcriptome similar to EIF2S2 and E2F3 knockdown cells (Fig. 4C).

### Resistant cells harbor mutations in other genes but VCP mutations are sufficient to produce resistance to CB-5803

**Figure 5:**
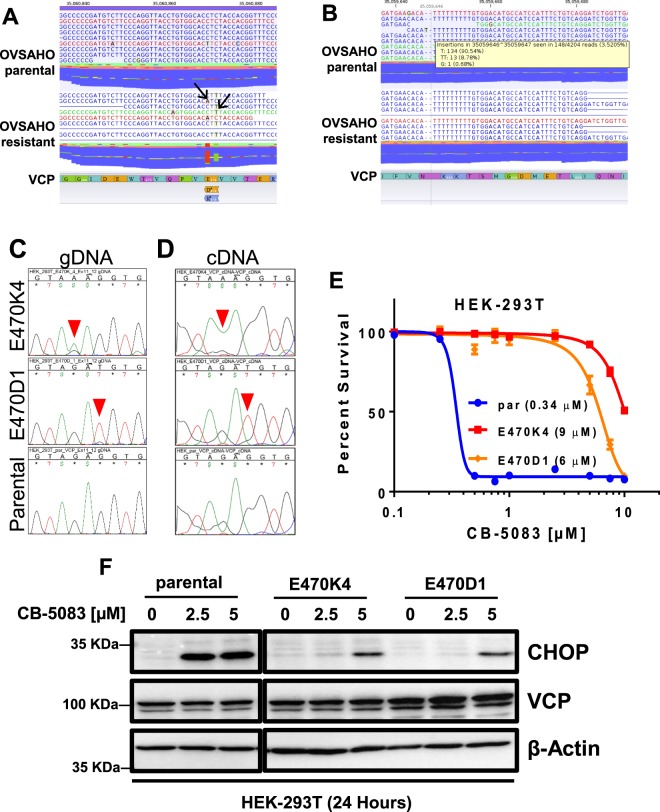
RNA-sequencing of parental and resistant OVSAHO cells indicate heterozygous mutations at codon 470 in resistant cells but not in parental cells. (**A**) Two different mutations (E470K4 and E470D1) in resistant cells. Mutated bases are indicated by arrows. (**B**) Less than 4% of transcripts (148 out of 4204 reads) contain insertional mutations at codon 616. (**C**,**D**) Genomic and cDNA sequencing indicates efficient editing of codon 470 by CRISPR-based gene editing. Mutated bases are indicated by the red arrowhead. (**E**) HEK-293T cells harboring observed mutations at codon 470 are more resistant than parental cell1. (**F**) Clone #4 harboring E470K (E470K4) and Clone #1 harboring E470D (E470D1) show attenuated UPR upon CB-5083 treatment. The western blot images were taken from the same blot but are cropped to remove the irrelevant lanes.

Our previous studies have shown that we can extract reliable mutational evidence from RNA sequencing studies^26^. Therefore, we analyzed the RNA sequence reads to identify mutations in parental and resistant cells. Consistent with our previously reported Sanger sequencing results^21^, we detected both E470K (C > T) and E470D (C > A) missense mutations in VCP transcripts from OVSAHO resistant, but not in parental cells (Fig. 5A). Furthermore, we detected low level (<4%) nonsense mutations at codon 603 (data not shown) and codon 616 in both parental and resistant transcripts (Fig. 5B), which further confirmed that transcripts with nonsense mutations at codon 603 and codon 616 were subjected to nonsense-mediated decay (NMD).

Subsequently, we performed mutational analysis to detect other nonsynonymous mutations in the resistant cell line compared to the parental cell line. We identified 71 unique nonsynonymous substitutions in resistant cells at read coverage of at least 25 × (Table S1). Metascape analysis of mutated genes observed in resistant cells indicates the enrichment of genes involved in protein catabolic process, protein stabilization, and HSF1 activation (Fig. S2). Analysis of pathways based on the Protein-Protein Interaction database through Metascape web resources indicates the enrichment of proteolysis and ubiquitin-dependent protein catabolic processes in genes mutated in resistant cells (Fig. S2). In Table 1, we selectively list 10 nonsynonymous substitutions in OVSAHO-R1. It should be noted that these mutations are found by comparing abundant transcripts between parental and resistant cells, and they likely represent true mutations that are not found in parental cells.

**Table 1:** Select non-synonymous mutations in resistant cells discovered by RNA sequencing.

Gene	Reference	Substitution	Zygosity
VCP	E470	K	Heterozygous
VCP	E470	D	Heterozygous
FLNA	R484	W	Heterozygous
UQCR10	S8	L	Heterozygous
MCM7	A241	S	Heterozygous
APBB2	T131	S	Homozygous
TNKS1BP1	P602	H	Heterozygous
ADAM10	I385	F	Heterozygous
SLC16A3	D260	A	Heterozygous
TRAK1	N857	S	Heterozygous
ATG4B	V364	D	Heterozygous

Considering that CB-5083-resistant OVSAHO cells harbor additional mutations, we next tested the extent to which the observed VCP mutations are sufficient in conferring resistance towards CB-5083. We performed CRISPR-Cas9 based *VCP* gene editing^27^ to produce both E470K and E470D mutants in HEK-293T cells. Genomic DNA (gDNA) and complementary DNA (cDNA) sequencing indicate efficient editing of the targeted codon (Fig. 5C,D). Both CRISPR edited HEK293T cell lines (E470K4 and E470D1) with mutated VCP show at least 12-fold higher resistance to CB-5083 compared to parental HEK-293T cells (Fig. 5E). These results suggest that observed mutations in VCP are sufficient to produce resistance to CB-5083. Consistent with our previous studies indicating that E470K and E4700D mutants have higher *in vitro* ATPase activity and require a higher concentration of CB-5083 to inhibit the enzyme activity, cells harboring these mutants show attenuation of the UPR when treated with CB-5083 at the concentrations that induced robust CHOP expression in parental cells (Fig. 5F).

### Resistant cells harbor heterozygous mutations in VCP allele but not in the VCP allele with a preexisting frameshift mutation

**Figure 6:**
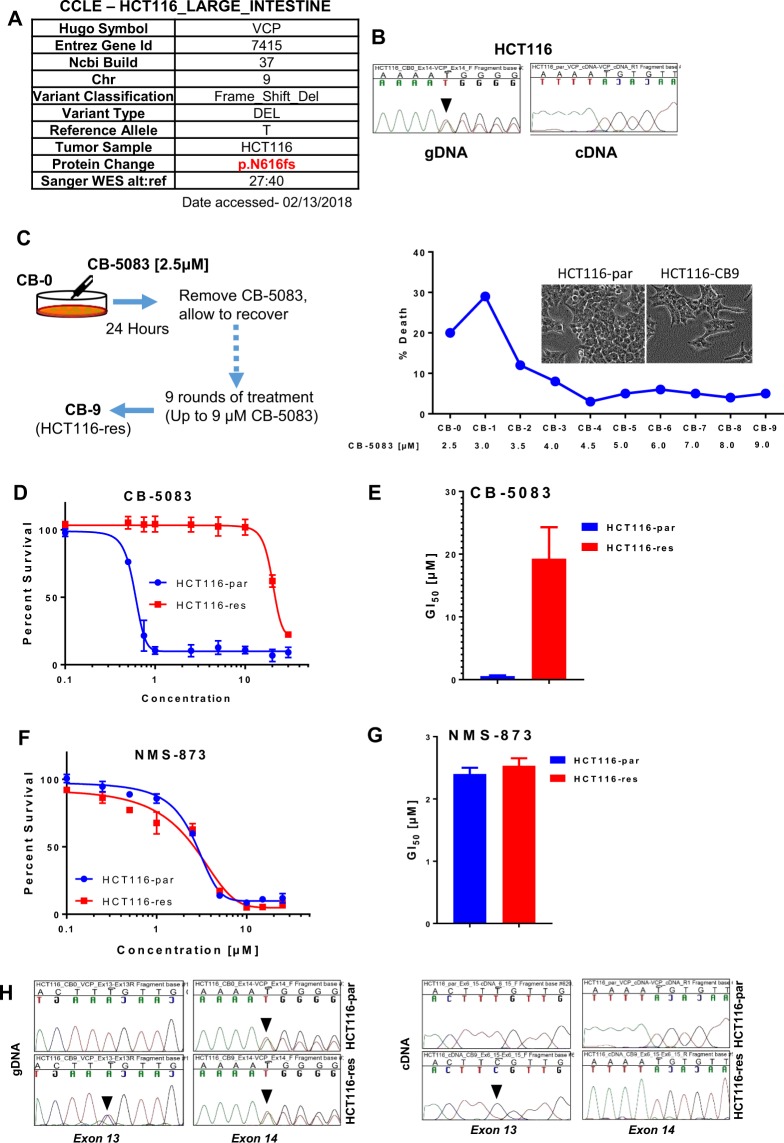
HCT116 harbor preexisting N616fs* mutation in one allele. (**A**) Analysis of HCT116 exome sequencing results from the Cancer Cell Line Encyclopedia (CCLE). (**B**) Chromatograms of the genomic DNA (gDNA) and complementary DNA (cDNA) sequences displaying partial sequences of *VCP* exon 16 in parental HCT116 cells. (**C**) Schematic showing the development of HCT116 resistance cell line (Also refer to Materials and Methods). (**D**) Parental HCT116 (HCT116-par) and CB-5083-resistant HCT116 (HCT116-res) cells were incubated with different doses of CB-5083 between 0.1 µM and 30 µM for 72 hours. Dose-response curves were generated with the Graph-Pad Prism using four parameters nonlinear regression and the curves were constrained at the top (100%) and the bottom (>0%). Each point in the dose-response curve represents Mean ± SEM from three technical replicates. (**E**) The bar-graph represents Mean GI_50_ + SEM obtained from three biological replicates. (**F**,**G**) Similar to (**C**,**D**) but treated with increasing doses of NMS-873 between 0.1 µM and 25 µM for 72 hours. (**H**) Chromatograms of the gDNA and cDNA sequences displaying specific regions of *VCP* in the HCT116-par and the HCT116-res cells. Black arrows point to the observed mutations in *VCP*.

Next, we looked to profile all mutations in *VCP* recorded in the cancer cell line encyclopedia (CCLE). In total, we found 98 cancer cell lines that harbored mutations in *VCP* among which N616fs* was the most frequently reported mutation in these samples (Supplementary Table S3). N616fs* is also the most frequently reported mutation in the cancer genome atlas (TCGA) tumor samples (Supplementary Table S4). During this analysis, we saw that HCT116 cells harbored a heterozygous frameshift mutation at codon 616 (N616fs*) (Fig. 6A). This observation is important because prior studies by Her *et al*.^20^ and Anderson *et al*.^16^ used HCT116 to generate clones that are resistant to VCP inhibitors. Both studies reported mutations in *VCP* contributing to resistance to VCP inhibitors CB-5083 and NMS-873. While Anderson *et al*. reported that their mutations in *VCP* are homozygous, Her *et al*. reported their mutation in VCP is heterozygous. Her *et al*. concluded that heterozygous mutation in *VCP* was sufficient for resistance to NMS-873. The presence of the preexisting frameshift mutation at codon 616 (N616fs*) could help explain the discrepancy observed in these two studies. To confirm N616fs* in HCT116 cells, we performed Sanger sequencing of genomic DNA (gDNA) flanking exon 14 of *VCP* that contains codon 616 sequences. The Sanger sequencing results confirm that HCT116 cells harbor a single-base deletion in codon 616 that causes a frameshift mutation (Fig. 6B). Given that the N616 frameshift mutation introduces a premature stop codon, which is 55 nucleotides upstream of the last exon-exon junction, we suspected that transcripts from the mutated allele will undergo NMD. To confirm this possibility, we performed Sanger sequencing of cDNA of *VCP* from HCT116. Not surprisingly, we did not observe the frameshift mutation at codon 616 in the cDNA sequencing analysis (Fig. 6B), confirming that transcripts from the mutated allele are subjected to NMD. Our results show that VCP inhibitor resistance can only be conferred through complete loss of the wild-type VCP allele.

**Figure 7:**
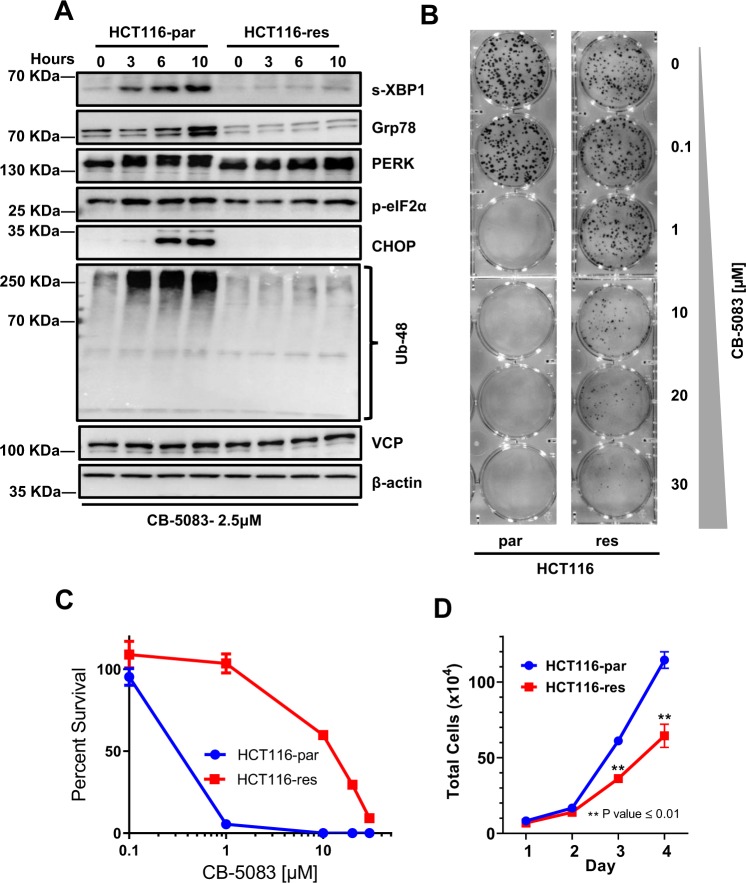
Resistant cells show attenuated UPR following CB-5083 treatment. (**A**) HCT116 parental (HCT116-par) and HCT116 resistant (HCT116-res) cells were treated with 2.5 µM of CB-5083. Cells were harvested at 3, 6 and 10 hours. 0 hour represents DMSO treated cells incubated for 10 hours. Protein lysates were probed against the indicated antibody. β-Actin acted as the loading control. (**B**) 1000 single cells (HCT116-parental and HCT116-res) were incubated with different concentrations of CB-5083 up to 30 µM for 48 hours. Cells were then allowed to recover in normal media for additional 6–10 days. Each dot represents a colony formed from the initial single cell. (**C**) The graph represents the calculated percent survival based on the absolute total colony count from three separate colony formation assays (7B). (**D**) Absolute live cell count of HCT116 parental (HCT-par) and HCT116 resistance (HCT116-res). Each data point represents the live cell count from three independent replicate samples.

To provide additional evidence that N616fs* allele is not subjected to additional mutational selection pressure under drug treatment, we independently generated CB-5083 resistant cells in HCT116 (HCT116-res) (Fig. 6C). HCT116-res cells displayed approximately 34-fold resistance towards CB-5083 (Fig. 6D,E) while displaying no cross-resistance towards NMS-873 (Fig. 6F,G). Resistance to CB-5083 was observed in a long-term colony formation assay as well (Fig. 7B,C). Subsequently, we sequenced the gDNA and cDNA to identify potential mutations in VCP. In HCT116-res cells, we identified a single heterozygous mutation (A > C) at codon 526 (L526S) in gDNA sequencing but observed a homozygous mutation (A > C) at codon 526 (L526S) in cDNA sequencing (Fig. 6H). These results suggest that the L526S mutation occurs in the allele that does not have the preexisting N616fs* mutation. These results also suggest that only the wild-type allele is subjected to a selection pressure under drug treatment in HCT116 cells.

### Cells with mutant VCP show increased resistance to CB-5083-induced unfolded protein response

To assess differences in the activation of the UPR by CB-5083 in the parental HCT116 and its resistant counterpart, we treated the cells with 2.5 *µ*M CB-5083, and induction of the UPR was monitored at various time points up to 10 hours. Within 3 hours of treatment, the UPR was activated in parental cells as evidenced by the induction of spliced XBP1 (s-XPB1), phosphorylated PERK, and phosphorylated eIF2a (Fig. 7A). Subsequently, in parental cells, CHOP expression was induced at 6 and 10 hours of CB-5083 treatment, and Grp78 was induced at the 10-hour time point. No such UPR activation was apparent in resistant cells. Consistent with the UPR activation in parental cells, CB-5083 induces an increase in ubiquitinylation at K48 in cellular proteins in parental cells but not in resistant cells. Additionally, HCT116-res cells display a significant decreased in proliferation compared to parental cells (Fig. 7D).

### N616fs* mutation is subjected to nonsense-mediated decay

**Figure 8:**
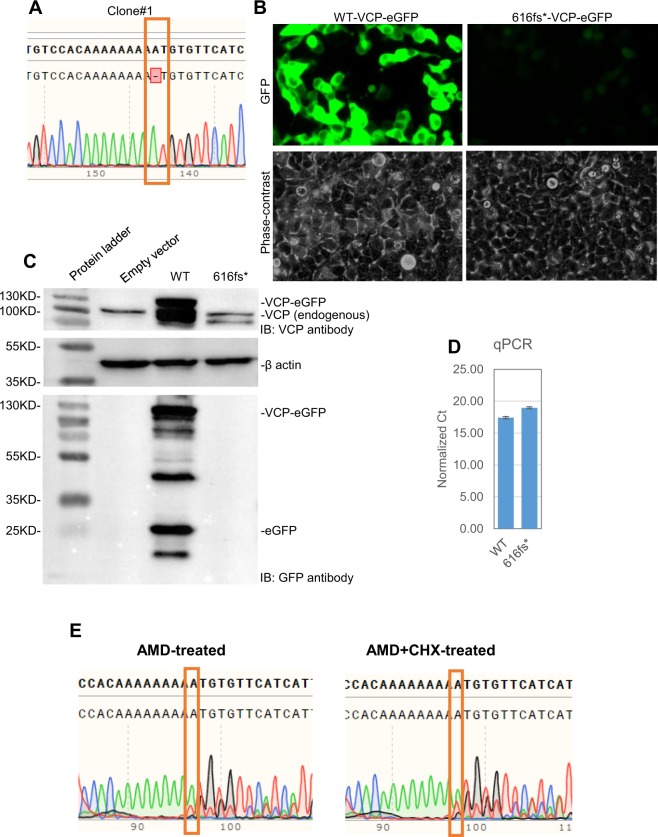
616 fs* mutation undergoes nonsense-mediated decay. (**A**) Sanger sequencing results show a single A deletion of the mutated plasmid that produces 616 fs*. (**B**) Transfection results indicate VCP-eGFP containing 616 fs* expressed at barely detectable levels. (**C**) Western blot analysis indicates robust expression of wild-type VCP-eGFP fusion protein but undetectable levels of VCP -eGFP with 616 fs* mutation. (**D**) Quantitative PCR of isolated DNA indicates similar levels of transfected plasmid, implying that reduced expression of VCP-eGFP with 616 fs* is not likely due to differences in transfection efficiency. (**E**) Sanger sequencing results indicate 616 fs* mutant transcript is partially stabilized by cycloheximide (CHX) plus actinomycin D (AMD) suggesting that it undergoes NMD.

Our sequencing data clearly show that the N616fs* VCP allele is expressed in low abundance, suggestive of NMD. To test this possibility, we obtained the wild-type VCP expression plasmid VCP(wt)-eGFP and performed site-directed mutagenesis at codon N616 to delete a single T to mimic the N616fs* mutant that we observed in HCT116 (Fig. 8A). The plasmid harbors a chimeric VCP-eGFP in-frame, therefore we expected the deletion to cause a loss of VCP and GFP expression. As expected, upon transient transfection we saw robust wild-type exogenous VCP and GFP expression as detectable by the western blot and GFP fluorescence (Fig. 8B,C), whereas the N616fs* mutant VCP and GFP expression is not detectable upon the transfection of the VCP N616fs*-eGFP plasmid. These differences in abundance between the wild-type vs the N616fs* plasmids are not due to transfection efficiency because quantitative qPCR of the transfected plasmids from genomic DNA extracts indicate comparable abundance (Fig. 8D).

NMD is a translation-dependent mechanism that degrades mRNA with premature stop codons. In order to confirm that the N616fs* mutant allele of HCT116 undergoes NMD, we treated cells with actinomycin D (5 μg/ml) only or actinomycin D and cycloheximide (100 μg/ml) for 2 hours, isolated RNA, and performed cDNA sequencing. The abundance of the mutant transcript in the actinomycin and cycloheximide D treated (AMD+CHX-treated) samples was increased compared to the actinomycin D treated (AMD-treated) samples (Fig. 8E), suggesting that the mutant allele is partially rescued by the treatment. These results further support our conclusions that N616fs* mutant is subjected to NMD.

## Discussion

We analyzed the transcriptome in the parental and the CB-5083 resistant cells upon CB-5083 treatment. The results show a strong induction of the unfolded protein response (UPR) in the parental cells further underscoring the on-target effect of CB-5083. Induction of the UPR was also observed in the resistant cells with 5 *µ*M of CB-5083 treatment suggesting target engagement at 5 *µ*M. Our gene set enrichment analyses show the downregulation of c-Myc targets upon CB-5083 treatment in parental cells. Recent publication unearthed a VCP-mediated Myc feedback mechanism, where VCP inhibition resulted in the accumulation of ubiquitinated c-Myc resulting in the downregulation of c-Myc target genes^28^. Our results provide further support for this mechanism.

In this study, we show that heterozygous mutations are not sufficient to cause resistance to VCP/p97 inhibitors. Previously reported heterozygous mutations, associated with resistance to VCP inhibitors in HCT-116, occurred in the context of preexisting heterozygous frameshift deletion in *VCP* at codon 616 (N616fs*). Here, we show that the N616fs* mutant undergoes NMD, and therefore the resistance-associated heterozygous mutant allele is the only copy that is expressed in these cells. Similarly, we also generated HCT116 resistant cells harboring a missense mutation (L526S) in *VCP* that resulted in resistance towards CB-5083. Moreover, this mutant does not contribute to cross-resistance with another VCP inhibitor NMS-873. During the preparation of this manuscript, an independent study identified L526S variant in CB-5083 resistant cells^29^.

More importantly, our results help to explain the differences in missense mutation zygosity reported by Anderson *et al*.^16^ and Her *et al*.^20^ in the same cell line HCT116. Based on these results, it is more than likely that the single heterozygous missense mutation (A530T) reported by Her *et al*. resides on the allele that does not have the preexisting N616fs* mutation and that their resistant cells solely express mutant VCP proteins. Authors should confirm the exclusive mutations in two *VCP* alleles by performing both complementary DNA sequencing and genomic DNA sequencing of their resistant cells. HCT116 cells harbor deficiency in mismatch repair^30^ as well as display low expression of P-glycoprotein^31^, which makes it an ideal cell line to screen for drug-resistant clones. It is therefore not surprising that HCT116 cells were used to screen for drug resistance. While Anderson *et al*. sequenced the cDNA to identify homozygous mutations in *VCP*, Her *et al*. sequenced the gDNA to observe the heterozygous mutation (A530T). By sequencing *VCP* at the gDNA and cDNA level, we were able to resolve the conflicting results obtained from these studies. Given that HCT116 cells harbor a preexisting N616fs* mutation that is subjected to NMD, reported studies including ours do not provide conclusive evidence that a single heterozygous mutation in *VCP* is sufficient to produce resistance to VCP inhibitors. Given that the functional unit of VCP is a homo-hexamer, a heterozygous mutation in *VCP* is expected to produce hexamers consisting of both wild-type and mutant VCP subunits. In such scenario, it will be important to determine if the inhibition of the wild-type subunits in the hexamers by VCP inhibitors can, in fact, compromise the function of the entire hexamer and contribute to sensitivity to VCP inhibitors. CRISPR/Cas9-mediated homology-directed repair can be used to correct the N616fs* mutation in HCT116 resistant cells to answer this important question. Interestingly, in our previous studies, we observed activating mutations (E470D and E470K) in one *VCP* allele and inactivating mutations (Q603* and N616fs*) in another *VCP* allele in OVSAHO ovarian cancer cells that were selected for resistance to CB-5083 (21). These results suggest heterozygous mutations in one *VCP* allele may not be sufficient to produce resistance to VCP inhibitors unless the other allele is subjected to inactivating mutations or perhaps separate activating mutations.

Given that VCP functions as a hexameric enzyme, the incorporation of a mutant unit to the hexamer could impact the function of the hexamer. Accordingly, mutations in *VCP* that are associated with multisystem proteinopathy are reported as heterozygous mutations^7,32–34^. In these diseases, mutant VCP appears to have a dominant negative effect on the function of hexameric VCP. In contrast, heterozygous mutations in *VCP* may not be sufficient to confer VCP inhibitor resistance. In a heterozygous setting, it is plausible that normal VCP subunits can be inhibited by VCP inhibitors, and such inhibition may compromise the function of hexameric VCP. In HCT116, due to the presence of pre-existing N616fs* mutation in one *VCP* allele, a heterozygous resistance-conferring mutation in the other allele could produce mutant-only hexamers as a result of the N616fs* allele undergoing NMD. Our experimental results confirm that N616fs* mutant is not detectable at the protein level. In a cell line model where both *VCP* alleles are wild-type, for example in OVSAHO ovarian cancer cells, resistant cells harbor two distinct *de novo* mutations in the corresponding VCP alleles. One *VCP* allele acquired a missense mutation that resulted in the increased ATPase activity and resistance to CB-5083 whereas the other allele acquired inactivating truncation mutations^21^. Collectively, these results highlight that zygosity of *VCP* mutations is different between multisystem proteinopathy and drug resistance.

Although clinical trials of CB-5083 for solid tumors and hematological malignancies were recently prematurely terminated due to the off-target effect on PDE6 resulting in ocular dysfunction, additional VCP inhibitors are being explored as clinical leads for cancer treatment. Given that different VCP inhibitors such as CB-5083 and NMS-873 display similar mechanism of drug resistance associated with on-target mutations in *VCP*, it is important to investigate mutations through sequencing of *VCP* for resistance mechanisms associated with new clinical leads. Such sequencing must be done both at the genomic and complementary DNA level to appropriately delineate the effects of homozygous or heterozygous VCP mutants in drug resistance.

## Supplementary information

Supplementary info
