# Supplementary material for: Heterozygous mutations in valosin-containing protein (VCP) and resistance to VCP inhibitors
Source: Sci Rep. 2019 Jul 29;9:11002. doi: 10.1038/s41598-019-47085-9 (PMC6662852; doi:10.1038/s41598-019-47085-9)
Supplement: Supplementary file 1 — Supplementary info [file 41598_2019_47085_MOESM1_ESM.pdf]

Supplemental information

Heterozygous mutations in valosin-containing protein (VCP) and resistance to VCP inhibitors

Prabhakar Bastola[1], Rabeya Bilkis[2], Cristabelle De Souza[2,3], Kay Minn[4], and Jeremy Chien[3*]

[1]Department of Laboratory Medicine, University of California San Francisco, 185 Berry Street, San Francisco, 94143, California, USA

[2]Division of Molecular Medicine, University of New Mexico Health Sciences Center, 915 Camino de Salud NE, Albuquerque, 87131, New Mexico, USA

[3]Department of Biochemistry and Molecular Medicine, University of California, Davis. 2700 Stockton Blvd. Sacramento, 95817, California, USA

[4]Department of Laboratory Medicine and Pathology, Mayo Clinic, 200 First Street SW, Rochester, 55905, Minnesota, USA

*Corresponding author; jrchien@ucdavis.edu;ORCID: 0000-0003-4744-8374

Supplemental information

Complete results of Metascape analysis are available at the following link:
https://osf.io/uzdbw/

Complete results of GSEA analysis are available at the following link:
https://osf.io/uzdbw/

Complete results of Connectivity Map analysis are available at the following link:
https://os f.io/uzdbw/

Complete results of Mutation analysis from RNA sequencing of resistant cells are available at the following link:
https://osf.io/uzdbw/

Supplementary Table S1

Supplementary Table S2

Supplementary Table S3

Supplementary Table S4

Supplementary Figure S1

Supplementary Figure S2

Supplementary Figure S3

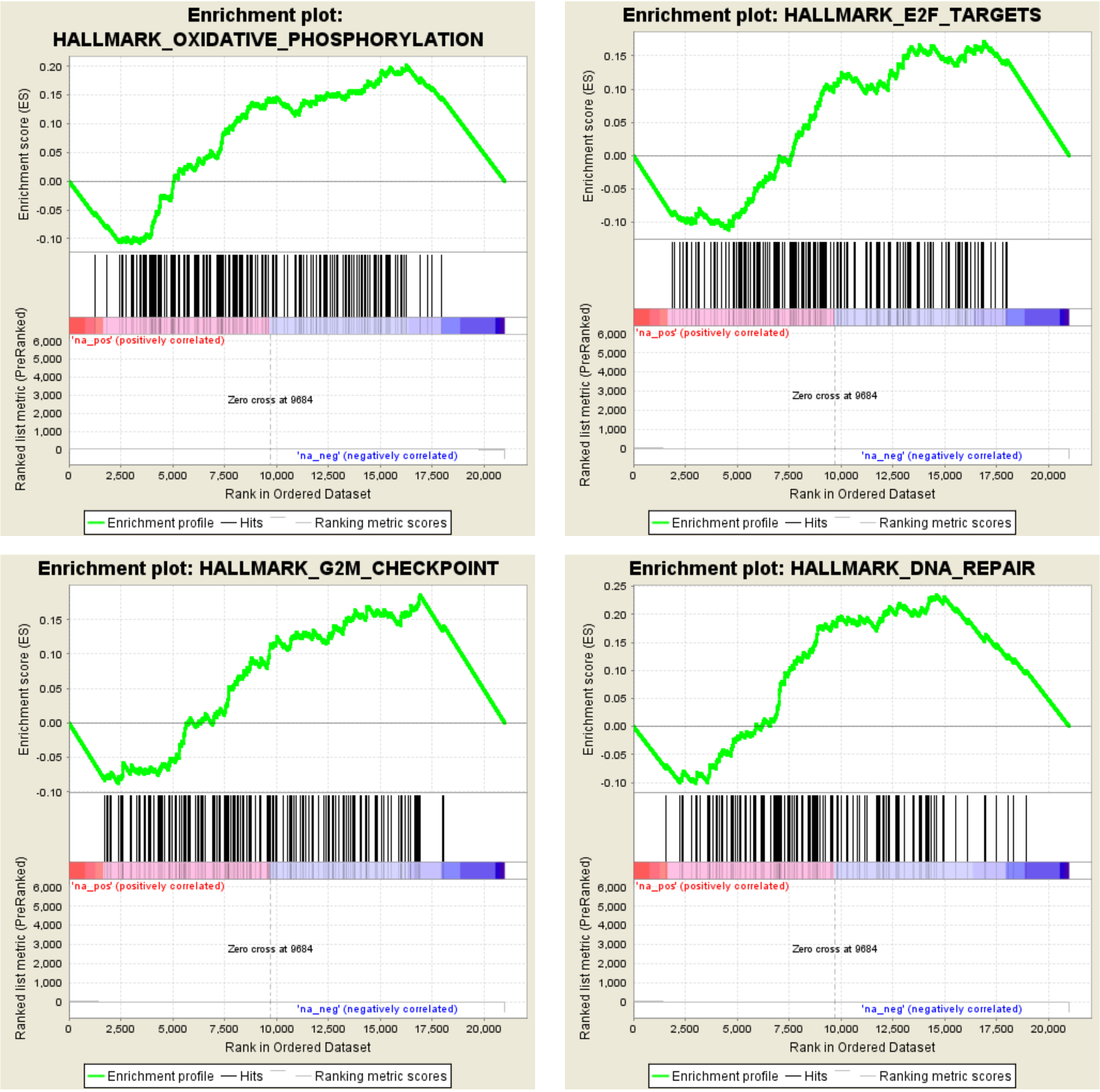

Figure S1: Hallmark gene sets positively enriched in resistant cells treated with CB-5083. Log2-transformed, ranked fold-change in gene expression between CB-5083-treated and DSMO-treated OVSAHO cells (CB/DMSO) is used for GSEA analysis. Na_pos = Genes upregulated by CB-5083. Na_neg = genes downregulated by CB-5083. Complete results of GSEA are available at the following link: https://osf.io/uzdbw/

A

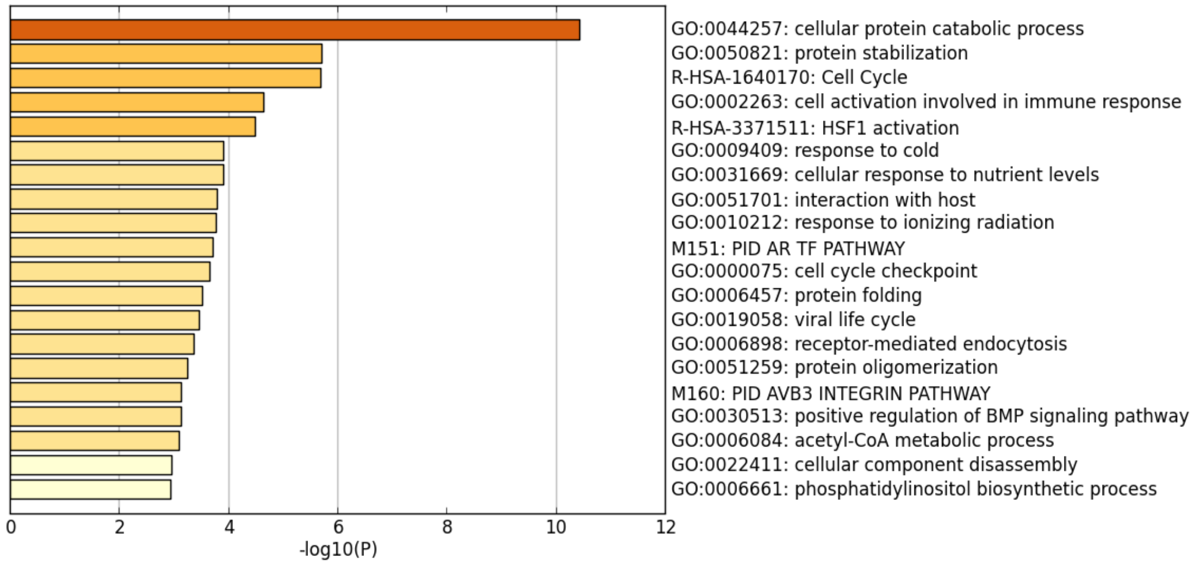

B KEGG Pathway, GO Biological Processes, Reactome Gene Sets, Canonical Pathways and CORUM

GO	Category	Description	Count	%	Log10(P)	Log10(q)
GO:0044257	GO Biological Processes	cellular protein catabolic process	22	18.03	-10.44	-6.13
GO:0050821	GO Biological Processes	protein stabilization	8	6.56	-5.70	-2.22
R-HSA-3371511	Reactome Gene Sets	HSF1 activation	3	2.46	-4.48	-1.45

C Protein-protein interaction network analysis

GO	Description	Log10(P)
GO:0044257	cellular protein catabolic process	-10.6
GO:0051603	proteolysis involved in cellular protein catabolic process	-10.0
GO:0006511	ubiquitin-dependent protein catabolic process	-9.8

E

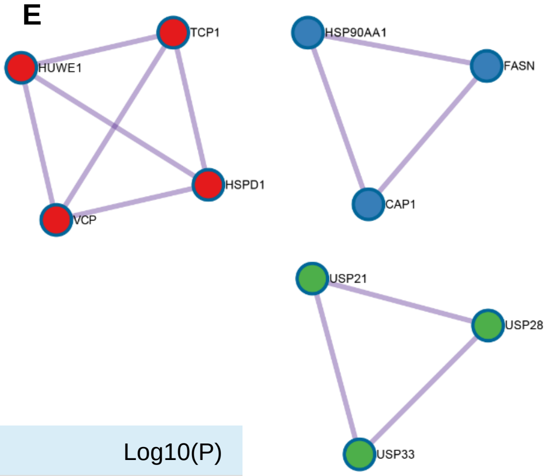

D MCODE network analysis

Color	MCODE	GO	Description	Log10(P)
(red)	MCODE_1	GO:0006457	protein folding	-5.4
(green)	MCODE_3	R-HSA-5689880	Ub-specific processing proteases	-6.0
(green)	MCODE_3	GO:0016579	protein deubiquitination	-5.7

Figure S2: Metascape analysis of mutated genes in CB-5083-resistant cells. (**A-B**) Gene Ontology (GO) Biological Processes, Reactome Gene Sets (R) and Canonical Pathways (M) significantly enriched by the mutated genes observed in resistant cells. (**C**) Protein-protein interaction networks enriched in mutated genes. (**D-E**). MCODE network analysis of genes mutated in resistant cells. Complete results of the Metascape analysis are available at the following link: https://osf.io/uzdbw/

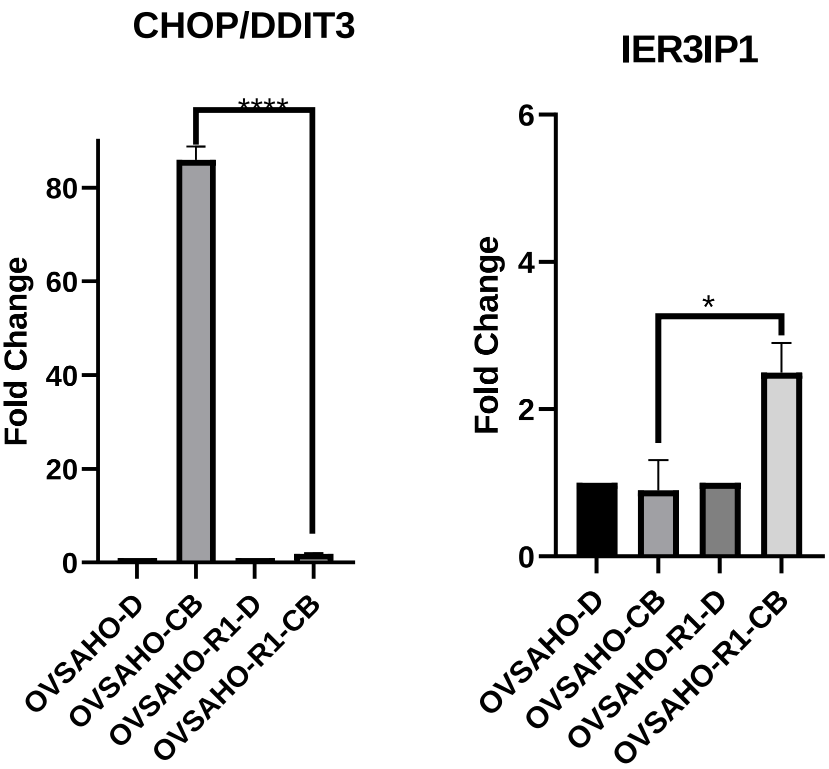

Figure S3: To assess the effect of CB-5083 treatment on OVSAHO Parental and OVSAHO-R1 cells, we performed RT-qPCR on both parental and resistant cell lines upon 6 hours of treatment with 5 µM CB-5083 (CB) or vehicle DMSO (D). Data are shown as mean ±.SEM. Statistics were performed with Student's t-test, and $p \leq 0.05$ was considered significant. * = $p \leq 0.05$, **** = $p \leq 0.0001$.

The following primers were used in the study:

CHOP

Forward	TGTTAAAGATGAGCGGGTGG
Reverse	CCTTCTTGAACACTCTCTCCTC

IER3IP1

Forward	CCTTTACCCTGTACTCACTGC
Reverse	CTGTGATTTAATTCCCGGCTC

GAPDH

Forward	GTCTCCTCTGACTTCAACAGCG
Reverse	ACCACCCTGTTGCTGTAGCCAA

Table S1.
The results are available at the following link:
https://osf.io/uzdbw/

The results are obtained from variant analysis of RNA sequencing data from parental OVSAHO vs resistant OVSAHO using CLC Genomic Workbench ver. 10. Only non-synonymous variants from the sequencing results at base positions with at least 25X coverage are shown.

Supplementary Table S1
https://osf.io/zaw79/

Supplementary Table S2
https://osf.io/r4y7v/

Supplementary Table S3
https://osf.io/nksmx/

Supplementary Table S4
https://osf.io/84e9t/
